# The Extract of *Rosa roxburghii* Tratt Alleviates Pulmonary Fibrosis in Mice via Gut Microbiota‐Amino Acid Metabolism and JAK2/STAT3 Inhibition

**DOI:** 10.1002/fsn3.71857

**Published:** 2026-05-03

**Authors:** Ting Zhou, Heting Zhou, Xinyue Zheng, Xiaomeng Wang, Tao Chen, Xingjie Li, Liqun Wang, Shouqian Li, Yongmei Xie, Lijun Peng, Wenya Yin

**Affiliations:** ^1^ West China School of Public Health and West China Fourth Hospital Sichuan University Chengdu Sichuan China; ^2^ State Key Laboratory of Biotherapy and Cancer Center, West China Hospital Sichuan University Chengdu Sichuan China; ^3^ Chengdu Institute of Product Quality Inspection Co., Ltd Chengdu China; ^4^ Department of Clinical Nutrition, Sichuan Provincial People's Hospital University of Electronic Science and Technology of China Chengdu China; ^5^ Guizhou Jinqianguo Biotechnology Co. Ltd. Bijie Guizhou China

**Keywords:** amino acid metabolism, gut microbiota, JAK2/STAT3, pulmonary fibrosis, *Rosa roxburghii* Tratt

## Abstract

Pulmonary fibrosis (PF) is an irreversible chronic lung disease in which dysregulation of tissue repair leads to excessive deposition of extracellular matrix (ECM). *Rosa roxburghii* Tratt (RRT), which has anti‐inflammatory and antioxidant properties, exhibits the potential to attenuate organ fibrosis. In this study, we evaluated the bioactive content and antioxidant capacity of different polar extracts of RRT (RRTEs), and explored the multi‐target mechanism of the optimal extract in alleviating PF. The ethyl acetate extract of RRT (EAE) exhibited the highest bioactive content and the strongest antioxidant capacity. EAE intervention reshaped the gut microbiota composition in PF mice by enriching beneficial bacteria and reducing pathogenic taxa. Metabolomic analysis identified 11 potential serum biomarkers associated with PF, which were involved in 7 metabolic pathways. Notably, EAE attenuated the disruption of L‐tryptophan metabolism, primarily through modulation of serotonin. Moreover, EAE was found to alleviate epithelial‐mesenchymal transition (EMT) and inhibit inflammatory cytokine via the JAK2/STAT3 pathway. In conclusion, EAE may exert anti‐PF effects associated with the structure of the gut microbiota, correction of amino acid metabolic disorders (notably tryptophan metabolism) and modulating JAK2/STAT3 signaling pathway in the lung. These findings provide new insights into the therapeutic potential of EAE against PF.

## Graphical abstract

The ethyl acetate extract of *Rosa roxburghii* Tratt (EAE) alleviated pulmonary fibrosis through modulation of gut microbiota‐amino acid metabolism and inhibition of the JAK2/STAT3 signaling pathway. Bioactive components and antioxidant capacity were evaluated across different polar extracts of *Rosa roxburghii* Tratt (RRTEs). The EAE exhibited the highest bioactive content and strongest antioxidant activity. In BLM‐induced pulmonary fibrosis mouse model, EAE treatment attenuated fibrotic pathology through integrated mechanisms. Specifically, EAE reshaped gut microbiota composition, corrected amino acid metabolic disturbances (particularly tryptophan metabolism), and suppressed JAK2/STAT3 pathway activation, leading to inhibition of epithelial‐mesenchymal transition (EMT). These findings suggest that EAE exerts anti‐fibrotic effects via multi‐target modulation along the gut‐lung axis.

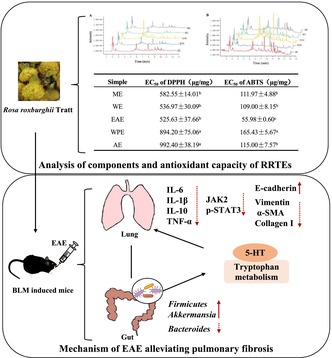

**Abbreviations**
5‐HT: 5‐hydroxytryptamine, Serotonin
AE: Ethanol extract of *Rosa roxburghii* Tratt
BLM: Bleomycin
EAE: Ethyl acetate extract of *Rosa roxburghii* Tratt
ECM: Extracellular matrix
EMT: Epithelial‐Mesenchymal Transition
LEfSe: Linear discriminant analysis Effect Size
ME: Methanol extract of *Rosa roxburghii* Tratt
PF: Pulmonary fibrosis
RRT:
*Rosa roxburghii* Tratt
RRTEs: Different polar extracts of *Rosa roxburghii* Tratt
WE: Water extract of *Rosa roxburghii* Tratt
WPE: The water‐phase extract left behind after ethyl acetate extraction of *Rosa roxburghii* Tratt

## 1 Introduction

Pulmonary fibrosis (PF) is a chronic, progressive lung disease characterized by damage to the lung and the development of scar tissue, accompanied by systemic metabolic disorders (Callaghan and Davenport Huyer 2025; Liu et al. 2022). The risk factors for PF include smoking, exposure to radiation, genetics, smoky environments, and infections (Suri et al. 2021). Furthermore, PF has emerged as one of the most devastating aftereffects of SARS, and COVID. The median survival rate of patients after diagnosis with PF is 3–5 years (Roque and Romero 2021), and the clinical manifestations are coughing, shortness of breath, and dyspnea. These symptoms have a significant negative influence on the quality of life and longevity of patients. Currently, nintedanib and pirfenidone are the clinical drugs predominantly used for the treatment of PF; however, these drugs can only slow the progression of the condition and exhibit unpleasant side effects, such as vomiting and gastrointestinal discomfort (Dempsey et al. 2019; Liu et al. 2022). Therefore, there is an urgent need for safe and effective new antifibrotic therapies. Recently, an increasing number of studies have focused on the application of natural products in PF treatment. Natural products with anti‐inflammatory and antioxidant properties have been considered potential alternative sources for preventing and treating PF (Chen et al. 2018; Hasan et al. 2022).

*Rosa roxburghii* Tratt (RRT) is favored by consumers because of its high nutritional value and health benefits and is widely grown in southwest China (He et al. 2016; Wang et al. 2023). Phytochemical studies have shown that RRT is rich in vitamins, amino acids, organic acids, polyphenols, flavonoids, triterpenes, and other nutrients, and its nutritional value is much higher than that of other common fruits, such as pomegranate and blueberries (Yang et al. 2020). As a high‐quality fruit used in medicine and food, RRT possesses excellent biological activities such as anti‐inflammation, anti‐oxidation, anti‐atherosclerosis, anti‐cancer, and regulation of metabolic disorders in diseases (Chen, Zhang, et al. 2024; Chen, Zhao, et al. 2024; Wang et al. 2021). Previous studies have shown that the polyphenols in RRT can inhibit inflammation and alleviate lipopolysaccharide‐induced acute lung injury (Tang et al. 2023). Additionally, *Rosa roxburghii* fermentation broth could alleviate pulmonary fibrosis, potentially via alleviating intestinal disorders (Zhou et al. 2025). However, fermented products contain a complex mixture of plant compounds and microbial metabolites, making it difficult to attribute the efficacy to specific phytochemicals. Furthermore, the regulatory effect of RRT with dietary therapeutic characteristics on metabolic disorders in PF mice has not yet been elucidated.

Therefore, this study aimed to perform a bioactivity‐guided fractionation of RRT to identify the extract with the highest antioxidant capacity and bioactive content, and to investigate its mechanism of action in improving disease metabolism. Given the restricted modalities of RRT application and the fact that different solvents and extraction methods affect the active components and their content in the extracts, resulting in varying biological effects. we extracted RRT using four solvents of different polarities. We then screened the RRT extract for optimal physicochemical properties and investigated its anti‐PF effects and underlying mechanisms based on the gut microbiota and serum metabolomics. This study provides theoretical guidance for the optimization of RRT extraction and its application in the prevention and treatment of PF.

## 2 Materials and Methods

### 2.1 Preparation of RRT Extracts

The RRT were grown in Anshun City, Guizhou Province, China. The RRT were washed, sliced, freeze‐dried, and milled into a fine powder. This powder was extracted twice using ultrasound and methanol, ethanol, and water (1:2, m/v) for 1 h. The filtrate was collected, concentrated using rotary evaporation, and freeze‐dried to obtain the methanol extract (ME), ethanol extract (AE), and water extract (WE). Ethyl acetate was then used to treat the WE before drying (2:1, v/v) to obtain two different extract fractions: the ethyl acetate extract fraction (EAE) and the water phase extract fraction (WPE). These extracts were then concentrated and freeze‐dried to obtain the powders of the different extract fractions. Different polar extracts of RRT (RRTEs) were stored at −80°C until use.

### 2.2 Analysis of Extract Composition

Sample Preparation: RRTEs were prepared separately in methanol to a concentration of 0.1 mg/mL, sonicated, and filtered into liquid phase vials for HPLC using Q‐Exactive Orbitrap MS (Thermo Fisher, Germany).

Liquid chromatography: Column temperature, 40°C; flow rate, 0.3 mL/min; mobile phase A, 0.1% formic acid aqueous solution; mobile phase B, acetonitrile. The gradient was set as follows: 0 min, 95% A; 0.5 min, 95% A; 3 min, 80% A; 6 min, 50% A; 8 min, 30% A; 8.5 min, 10% A; 10 min, 10% A; 10.1 min, 95% A.

Mass spectrum: The ion source type of mass spectrometry was electrospray ionization (ESI), and the detection mode used was full‐ddMS2 mode. The parameters were as follows: positive ion spray voltage, 3.5 kV; negative ion spray voltage, 2.5 kV; sheath gas, 50 Arb; auxiliary gas, 15 Arb; sweep gas, 2 Arb; ion transfer tube temperature, 320°C; vaporizer temperature, 350°C.

### 2.3 Determination of Total Polyphenols, Flavonoids, and Triterpenes

Total polyphenols, flavonoids, and triterpenes were determined using a previously described method, with slight modifications (He et al. 2016; Qneibi et al. 2021). The total polyphenol content was determined using the Forintol method, and the absorbance at 765 nm was measured using gallic acid (Shanghai Yuanye Biotechnology Co. Ltd., China) as a standard. Total flavonoid content was determined using the NaNO_2_‐Al(NO_3_)_3_‐NaOH method, and the absorbance at 510 nm was measured using rutin (Shanghai Yuanye Bio‐Technology Co. Ltd., China) as a standard. The total triterpene content was determined, and the absorbance at 548 nm was measured using ursolic acid (Shanghai Yuanye Bio‐Technology Co. Ltd., China) as a standard.

### 2.4 Determination of Antioxidant Capacity

The antioxidant capacity of RRTEs was evaluated by determining the DPPH and ABTS free radical scavenging capacities, as described in a previous study (Wang et al. 2022).

### 2.5 Animals

All animal experiments complied with the guidelines of the National Institutes of Health (NIH) and were approved by the Ethics Committee of the Fourth West China Hospital of Sichuan University and the West China School of Public Health [license number: Gwll2022105]. Male C57BL/6 mice (7–8 weeks old) were purchased from Beijing Vital River Laboratory Animal Technology Co. Ltd. (Beijing, China) and raised in an SPF environment. After 1 week of acclimatization, the mice were randomly divided into control, model, EAE, and negative control (NC) groups (*n* = 10). Mice in the control and NC groups were injected with normal saline into the trachea, and mice in the remaining two groups were injected with 2 mg/kg BLM into the trachea to induce PF. One day after the injection, 400 mg/kg of the corresponding EAE was administered to mice in the EAE and NC groups, and equal amounts of saline were administered to the control and model groups. The mice were sacrificed 21 days after their eyeballs were removed for blood collection. The upper end of each lobe of the lung was fixed in tissue fixative, and the rest of the lung tissues were snap‐frozen in liquid nitrogen and stored at −80°C.

### 2.6 Histopathological Analysis

After the fixed lung tissues were embedded in paraffin and sectioned, pathological changes in lung tissues were evaluated by hematoxylin–eosin staining (H&E) and Masson staining (Biossci Biotechnology Co. Ltd., China). Immunohistochemistry (Biossci Biotechnology Co. Ltd., China) was used to observe Collagen I, α‐SMA, and E‐cadherin expression.

### 2.7 Gut Microbiota Analysis

Mouse feces were collected and evaluated by Suzhou PANOMIX Biomedical Tech Co. LTD (Chengdu, China). The most suitable total DNA extraction method was selected, and the DNA was quantified using a Nanodrop. Amplification and purification of the target fragments were performed, followed by fluorescence quantification of the recovered PCR amplification products. Sequencing libraries were prepared using an Illumina TruSeq Nano DNA LT Library Prep Kit. High‐throughput sequencing was performed after quality inspection of the library. The library and samples were divided according to index and barcode information, and the barcode sequences were removed. Sequence denoising or OTU clustering was performed according to the QIIME2 Dada2 analysis process or the VSearch software analysis process. Further intergroup difference analyses were performed according to ASV/OUT.

### 2.8 Serum Metabolomics

Serum samples were pretreated with methanol‐acetonitrile and analyzed using UHPLC (1290 Infinity LC, Agilent Technologies) coupled to a quadrupole time‐of‐flight (AB Sciex TripleTOF 6600) at Shanghai Applied Protein Technology Co. Ltd.

Samples were analyzed using a 2.1 mm × 100 mm ACQUIY UPLC BEH Amide 1.7 μm column (Waters, Ireland). In both ESI positive and negative modes, the mobile phase contained 25 mM ammonium acetate and 25 mM ammonium hydroxide in water and acetonitrile. The gradient was 95% B for 0.5 min and was linearly reduced to 65% in 6.5 min, reduced to 40% in 1 min, maintained for 1 min, and then increased to 95% in 0.1 min, with a 3‐min re‐equilibration period. The ESI source conditions were as follows: ion source gas1, 60; ion source gas2, 60; curtain gas, 30; source temperature, 600°C; IonSpray Voltage Floating, ±5500 V. Raw MS data were converted to MzXML files using ProteoWizard MSConvert before being imported into freely available XCMS software.

### 2.9 Quantitative Real‐Time PCR (qPCR)

Total RNA in the tissue was extracted using an RNA extraction kit (Chengdu Foregene Company Limited, China), and cDNA was generated using a reverse transcription kit (ABclonal Technology Co. Ltd., Wuhan, China). Real‐time fluorescence quantitative PCR was performed using a Taq Pro Universal SYBR qPCR Mix Kit (Vazyme Biotech Co. Ltd., Nanjing, China) according to the manufacturer's instructions. The 2^−∆∆CT^ formula was used to analyze gene expression relative to GAPDH expression. Primers were synthesized by Sangon Biotech (Shanghai, China) and are listed in Table S1.

### 2.10 Western Blot

Tissues were lysed using a radioimmunoprecipitation assay (RIPA, Beyotime, China) and quantified using bovine serum albumin (BSA) as a standard. After separation by sodium dodecyl sulfate–polyacrylamide gel electrophoresis (SDS‐PAGE), the proteins were transferred onto polyvinylidene fluoride (PVDF) membranes. These protein membranes were blocked with 5% skim milk at room temperature for 1 h and incubated overnight at 4°C in primary antibodies at different dilution ratios. After three washes with Tris‐buffered saline containing Tween (TBST), the membranes were incubated with the secondary antibody for 1 h. Finally, proteins were detected using an enhanced chemiluminescence (ECL) kit and quantified using ImageJ.

### 2.11 Statistical Analysis

The results are presented as “mean ± standard deviation” of at least three independent experiments. Statistical analyses and graphing were performed using Excel, ImageJ, and GraphPad Prism 9. One‐way ANOVA and *t*‐tests were used to analyze differences between groups. Different lowercase letters indicate significant differences (*p* < 0.05) between groups, where * denotes a comparison with the control group and # denotes a comparison with the model group: *p < 0.05, **p < 0.01, ***p < 0.001, ^#^ *p* < 0.05, ^##^ *p* < 0.01, ^###^ *p* < 0.001.

## 3 Results

### 3.1 Analysis of the Antioxidant Capacity of RRTE Components

#### 3.1.1 Qualitative Analysis of RRTEs

**FIGURE 1:**
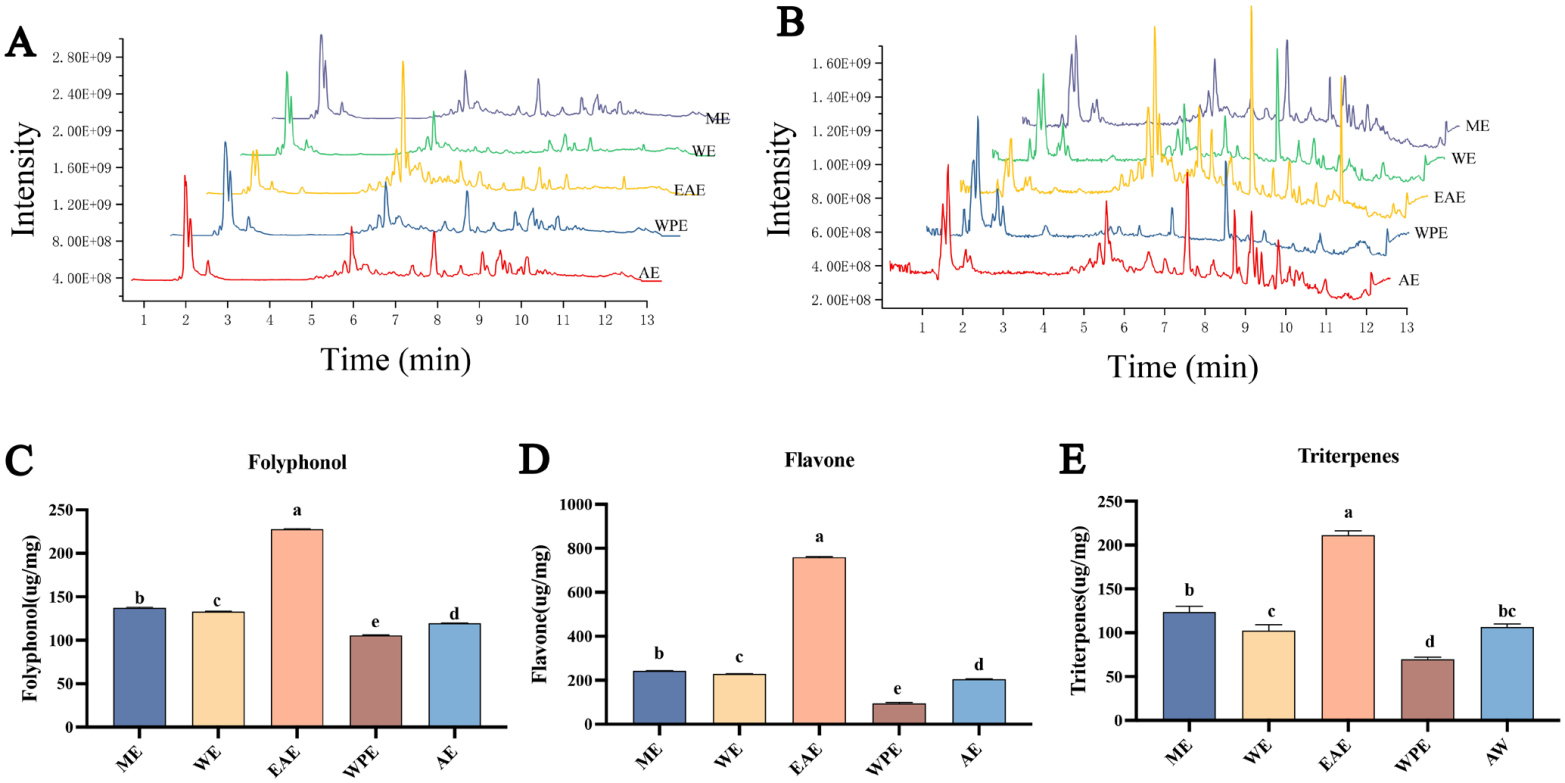
Analysis of the antioxidant capacity of RRTE components. Ion flow diagrams of the RRTEs in (A) negative ion mode and (B) positive ion mode. Contents of (A) total polyphenols, (B) total flavonoids, and (C) total triterpenes in RRTEs. Different lowercase letters between groups indicate significant differences *p* < 0.05.

HPLC‐Q‐Exactive Orbitrap‐MS was used to detect the possible components of the RRTEs, and the ion flow diagrams are shown in Figure 1A,B. A total of 41 components were obtained under the experimental conditions listed in Table S2. The bioactive substances included 10 polyphenols, 8 flavonoids, 9 organic acids, 6 glycosides, 4 amino acids, 2 triterpenoids, 1 polysaccharide, and 1 alkaloid. Through a lateral comparison of the peak areas of the components, the content of most bioactive substances in the EAE, such as proanthocyanidins, rosamultin, and epigallocatechin, was found to be higher than that in the four other extracts. This indicates that the EAE may retain more bioactive components of RRT.

#### 3.1.2 Bioactive Substances and Antioxidant Capacity of RRTEs

Polyphenols, flavonoids, and triterpenes are important components of RRT. Therefore, the total polyphenol, flavonoid, and triterpene contents in the different polar extracts of RRT were determined by colorimetry. As shown in Figure 1C–E, the total polyphenol contents of ME, WE, EAE, WPE, and AE were 137.16 ± 0.86, 132.96 ± 13.30, 227.56 ± 0.80, 105.76 ± 0.49, and 119.63 ± 0.20 μg/mg, respectively. The contents of total flavonoids were 363.91 ± 1.82, 344.82 ± 0.91, 759.97 ± 2.92, 143.61 ± 6.19, and 303.33 ± 2.62 μg/mg, respectively. The total triterpene contents were 123.73 ± 6.51, 106.44 ± 3.60, 102.39 ± 6.95, 211.29 ± 4.92, and 69.78 ± 2.24 μg/mg, respectively. The total polyphenol, flavonoid, and triterpene contents in EAE were significantly higher than those in WE, WE, WPE, and AE (*p* < 0.05). Thus, the highest contents of total polyphenols, flavonoids, and triterpenes in active substances were found in the EAE, indicating that EAE could enrich more bioactive substances.

The excellent antioxidant capacity of plants plays an important role in their defense against diseases. Therefore, we evaluated the antioxidant capacity of the five RRTEs using DPPH and ABTS free radical scavenging assays. Compared to the other extracts, EAE had a stronger scavenging ability for DPPH and ABTS radicals, with the smallest EC_50_ (Table 1). The EC_50_ of EAE for ABTS radical was 55.98 ± 0.60 μg/mL, which was significantly lower than that of the other extracts (*p* < 0.05). These results suggest that EAE may have health‐protective effects. Therefore, we explored the role of EAE in alleviating BLM‐induced PF in mice.

**TABLE 1:** Half scavenging capacity of RRTEs against DPPH and ABTS radicals.

Simple	EC_50_ of DPPH(μg/mg)	EC_50_ of ABTS(μg/mg)
ME	582.55 ± 14.01^b^	111.97 ± 4.88^b^
WE	536.97 ± 30.09^b^	109.00 ± 8.15^b^
EAE	525.63 ± 37.66^b^	55.98 ± 0.60^c^
WPE	894.20 ± 75.06^a^	165.43 ± 5.67^a^
AE	992.40 ± 38.19^a^	115.00 ± 7.57^b^

*Note:* Different lowercase letters indicate significant differences (*p* < 0.05) between group.

### 3.2 EAE Alleviates BLM‐Induced PF in Mice

**FIGURE 2:**
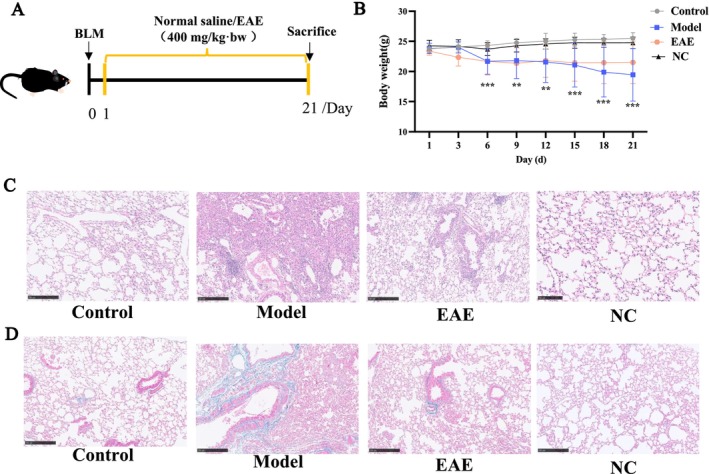
EAE alleviated BLM‐induced PF in mice. (A) Animal experimental design, (B) body weight of mice, (C) HE staining of lung tissue, magnification: 10×, (D) masson staining of lung tissue, magnification: 10×, Data are presented as mean ± SD. *p < 0.05; **p < 0.01; ***p < 0.001 compared to the Control; #*p* < 0.05; ##*p* < 0.01; ###*p* < 0.001 compared to the Model.

A BLM‐induced mouse model of PF was used to investigate the effects of EAE. As shown in Figure 2B, the weight of the control mice showed a continuous increase during the experimental period, and the body weight of mice in the model group continued to decrease compared with that of the control group and showed significant differences. Interventions with EAE were able to slow BLM‐induced body weight loss in mice. Furthermore, H&E staining (Figure 2C) showed significant inflammatory cell infiltration, alveolar wall thickening, alveolar lumen shrinkage, and formation of fibrotic masses in the lung tissues of the model group, suggesting that the PF model was successfully established. EAE ameliorated BLM‐induced lung injury in mice by reducing inflammatory cell infiltration and inhibiting fibrous scarring. Masson's trichrome staining (Figure 2D) also showed that EAE reduced collagen deposition in lung tissue. Additionally, the body weight and lung tissue structural characteristics of mice in the NC group were consistent with those of mice in the control group, indirectly indicating that EAE did not have detrimental effects on mice.

### 3.3 EAE Could Modulate Gut Microbiota Disorders in Mice With PF

**FIGURE 3:**
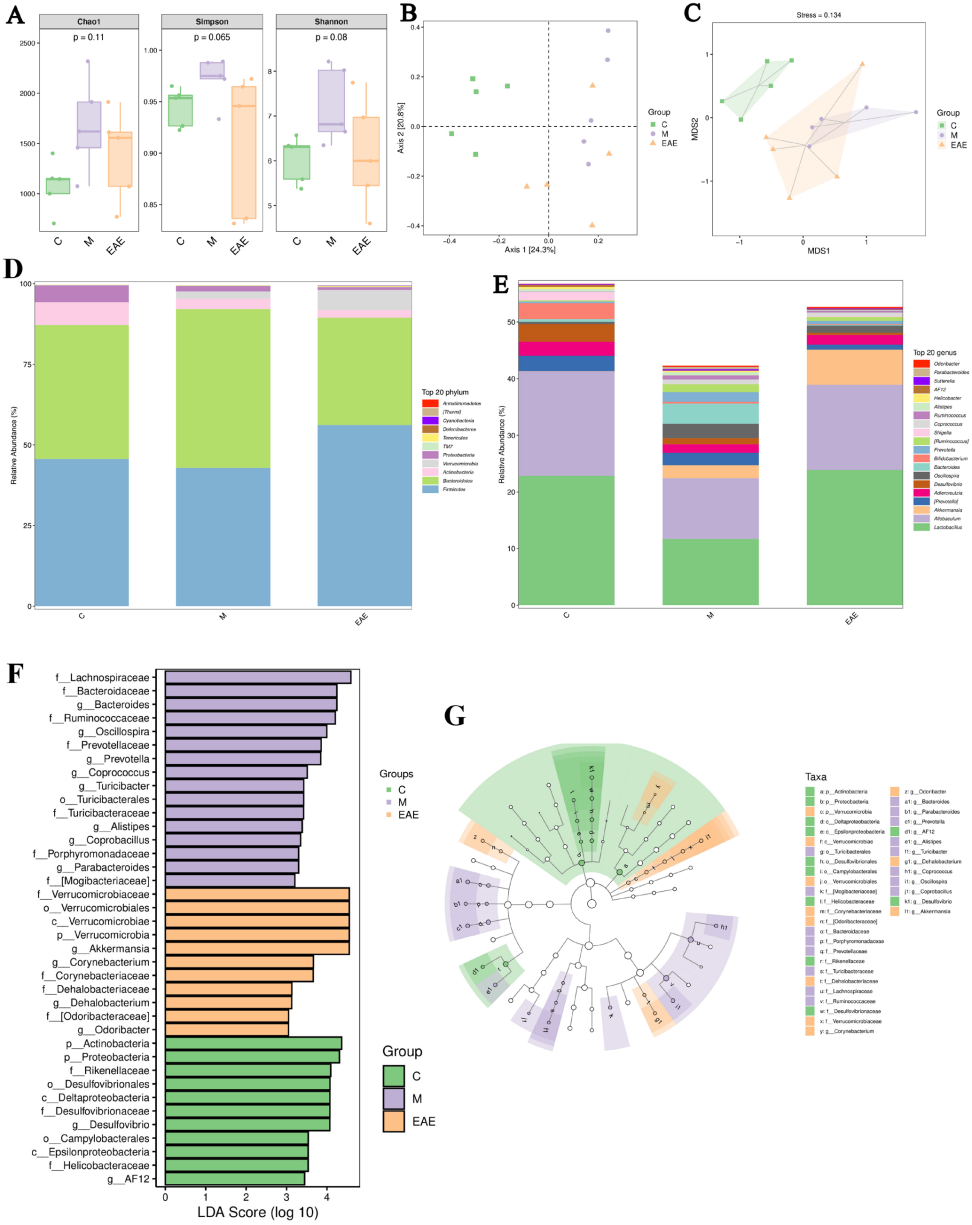
Effect of EAE on gut microbiota of mice with BLM‐induced PF. (A) Chao1, Simpson, and Shannon indices, (B) NMDS, (C) principal coordinate analysis PCoA, (D) bacterial composition at phyla level, (D) bacterial composition at genus level, (F) LDA score, and (G) taxonomic branching map of species.

To further explore whether RRT was involved in regulating the gut microbiota of mice with PF, we performed 16S rRNA amplicon sequencing. Chao1, Simpson, and Shannon indices were used to assess the alpha diversity of the gut microbiota. The results showed no significant differences in gut microbiota diversity between the groups (Figure 3A). We then used principal coordinate analysis (PCoA) and non‐measured multidimensional scaling (NMDS) to calculate the structural differences in gut microbiota between samples and assessed their β‐diversity. As shown in Figure 3B,C, the samples of the model group were clearly separated from the control group, and the sample points of the EAE group were also separated from those of the model group and in favor of the control group. The smaller the value of the stress, the better. The NMDS analysis results are considered more dependable when the value is less than 0.2. A stress of 0.134 for the NMDS in this experiment indicated that the data were stringent. Based on the above results, EAE may regulate intestinal bacteria disorders caused by BLM‐induced PF.

We further compared the relative abundance of gut microbiota between groups to assess the microbiota structure. At the phylum level, the relative abundance of *Firmicutes* decreased, and the relative abundance of *Bacteroidetes* increased in the model group compared with the control group; the EAE intervention increased the relative abundance of *Firmicutes* and decreased the relative abundance of *Bacteroidetes*, with a trend consistent with that of the control group (Figure 3D). At the genus level (Figure 3E), the relative abundance of *Lactobacillus*, *Allobaculu*, *Adlercreutzia*, *Bifidobacterium*, and *Shigella* was reduced in the model group compared to that in the control group, and *Oscillospira*, *Bacteroides*, *Prevotella*, and *Ruminococcu* increased in relative abundance. Interestingly, the EAE intervention mitigated the changes in the *Lactobacillus*, *Allobaculum*, *Adlercreutzia*, *Oscillospira*, and *Bacteroides* genera, bringing their relative abundances closer to those of the control. We then utilized linear discriminant analysis effect size (LEfSe) to further characterize the differential species of the gut microbiota in different groups. Figure 3F showed a bar graph of the distribution of the linear discriminant analysis (LDA) values, demonstrating that the species were significantly enriched within each group and their level of importance. Gut microbiota with LDA effect sizes > 2 and *p*‐values < 0.05 were considered statistically significant. Figure 3G showed the species taxonomic branching diagram (ladogram) and a hierarchical representation of the differential bacteria obtained from the LEfSe analysis. The control, model, and EAE groups comprised 11, 16, and 11 dominant communities, respectively. The dominant bacteria in the control group were *Actinobacteria* and *Proteobacteria*. The dominant flora in the model group were *Bacteroides, Turicibacter*, and other genera, whereas the dominant bacteria in the EAE group changed to *Verrucomicrobia* and *Akkermansia*. These findings suggest that the EAE intervention can regulate the structural disorder of the intestinal bacteria in BLM‐induced PF mice, increase beneficial bacteria, and produce healthy effects in PF mice.

### 3.4 Effects of EAE on Serum Metabolism

**FIGURE 4:**
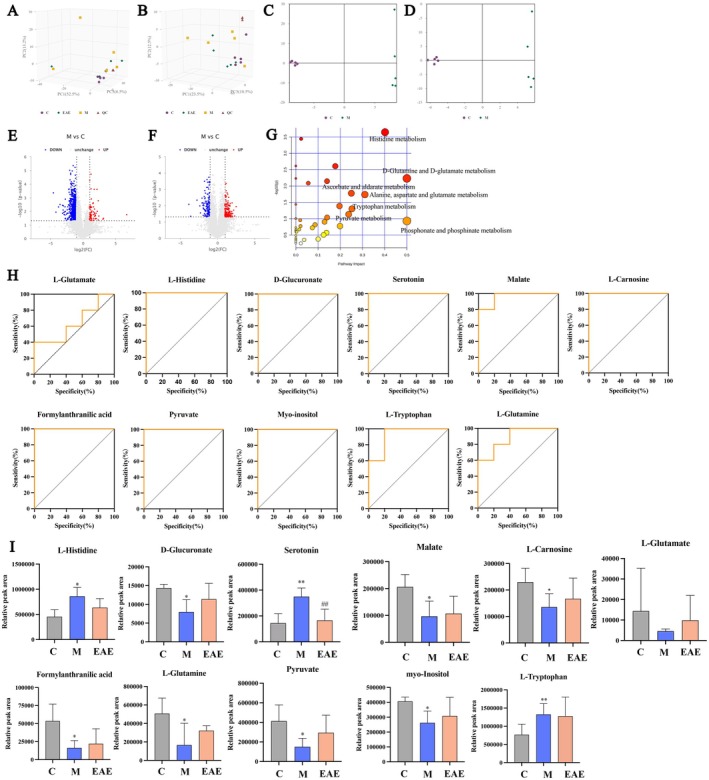
Effects of EAE on serum metabolism. PCA score plots of Control, Model and EAE groups in (A) negative ion mode and (B) positive ion mode, OPLS‐DA score plots of Control and Model groups in (C) negative ion mode and (D) positive ion mode, Volcano plots for Control and Model groups in (E) negative ion mode and (F) positive ion mode, (G) metabolic pathways summarized from serum differential metabolites between control and model groups, (H) ROC curves based on selected key biomarkers, (I) relative levels of key metabolites in serum samples. Data are presented as mean ± SD. *p < 0.05; **p < 0.01; ***p < 0.001 compared to the Control; #*p* < 0.05; ##*p* < 0.01; ###*p* < 0.001 compared to the Model.

To elucidate the metabolic pathways involved in the alleviation of PF by EAE, we performed a metabolomic analysis of serum samples using LC–MS. Serum samples from each group were analyzed using principal component analysis (PCA) in both positive and negative ion modes and quality control (QC). As shown in Figure 4A,B, QC sample points (represented by triangular symbols) in the PCA plot were closely clustered in both positive and negative ion modes, indicating centralized QC and instrument stability. We then used Orthogonal Partial Least Squares‐Discriminant Analysis (OPLS‐DA) to determine the differences in metabolites between the model and control groups (Figure 4C,D). The two groups were significantly separated, and intragroup aggregation reflected the differences between the groups.

Metabolites with VIP > 1 were considered to have a significant contribution to the model interpretation. We then analyzed the differential metabolites using strict OPLS‐DA VIP > 1 and *p* value < 0.05 as screening criteria to analyze the differential metabolites. Volcano plots of the differential metabolites are shown in Figure 4E,F and specified in Table S3. Compared with the control group, 42 substances were upregulated, and 71 substances were downregulated in the model group; thus, the serum metabolism of the model group was altered to a certain extent compared with the control group. EAE corrected 107 substances of the BLM‐induced differential metabolite disorders, of which 15 substances were statistically significant, suggesting that EAE has the potential to correct metabolic disorders in PF.

To understand the metabolic pathways affected by PF and obtain potential biomarkers, we introduced differential metabolites from the control and model groups into MetaboAnalyst 5.0 for metabolic pathway analysis, as shown in Figure 4G. Seven metabolic pathways, including the histidine, D‐glutamine and D‐glutamate, phosphonate and phosphinate, alanine, aspartate, and glutamate, ascorbate and aldarate, tryptophan, and pyruvate metabolism (impact > 0.2) were changed in BLM‐induced mice. Finally, 11 potential biomarkers were identified from these seven metabolic pathways (Table S4): L‐glutamate, N‐methylhistamine, L‐histidine, carnosine, L‐glutamine, CMP‐2‐aminoethylphosphonate, pyruvate, myo‐inositol, D‐glucuronate, L‐tryptophan, serotonin (also known as 5‐hydroxytryptamine, 5‐HT), formylanthranilic acid, and (S)‐malate. Receiver operating characteristic (ROC) curves were used to assess the diagnostic potential of these 11 potential biomarkers (Figure 4H). The results show that, except for L‐glutamine (area under the curve, AUC = 0.64), the remaining 10 biomarkers exhibited AUC values greater than 0.85, were top‐ranked candidates, and were determined to be promising biomarkers for PF diagnosis. Interestingly, compared with the model group, the EAE intervention in mice with PF seemed to regulate these biomarkers, but only 5‐HT in tryptophan metabolism was statistically significant (Figure 4I). According to the above results, all seven metabolic pathways are important in the action of RRT administration, and the tryptophan metabolism pathway may be the main metabolic pathway that plays an anti‐PF role. We found that EAE exerts its effects through 5‐HT.

### 3.5 Correlations Between Biomarkers of PF and Gut Microbiota

**FIGURE 5:**
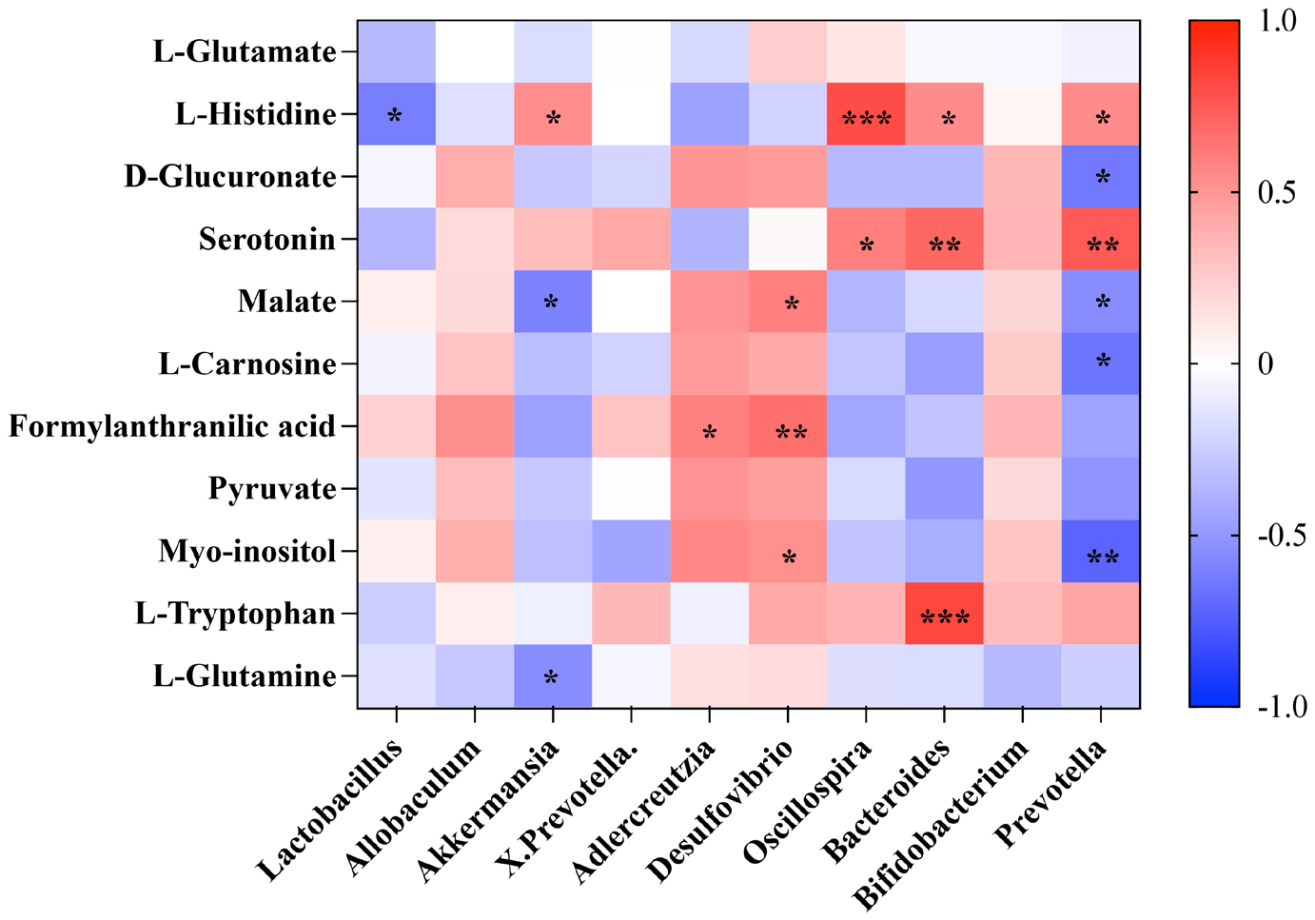
Spearman correlation analysis between key biomarkers and gut microbiota. *p < 0.05; **p < 0.01; ***p < 0.001.

To further explore the functional correlation between gut microbiota and metabolites in PF mice, we established a correlation analysis diagram by calculating the Spearman correlation coefficient. Figure 5 shows the correlations of the 11 potential serum biomarkers with the top 10 key gut microbiota at the genus level. The results show that nine potential biomarkers were significantly associated with the intestinal bacteria. For example, L‐histidine was negatively correlated with *Lactobacillus* abundance, and D‐glucuronate, malate, L‐carnosine, and myo‐inositol were negatively correlated with *Prevotella*. Additionally, L‐histidine and 5‐HT were positively correlated with *Prevotella* and *Oscillospira*, and formylanthranilic acid was positively correlated with *Allobaculum*. Furthermore, myo‐inositol was positively correlated with *Adlercreutzia*. Host metabolism and the intestinal bacteria structure are mutually affected, and disturbances in the microenvironment may affect the occurrence of diseases.

### 3.6 EAE Ameliorates PF by Suppressing Inflammation, JAK2/STAT3 Signaling, and EMT

#### 3.6.1 EAE Regulates BLM‐Induced Proinflammatory Cytokines

**FIGURE 6:**
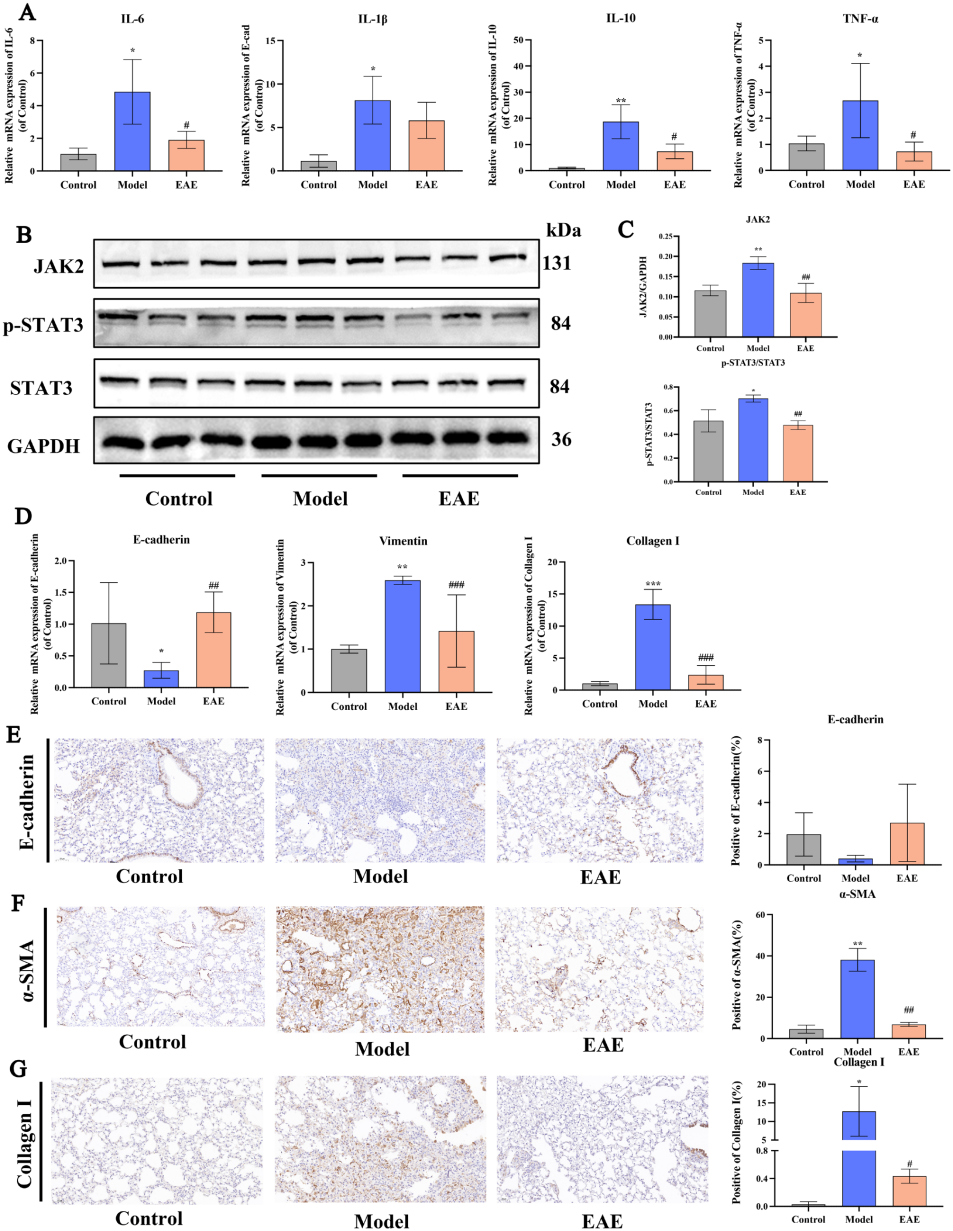
EAE ameliorates PF by suppressing inflammation, JAK2/STAT3 signaling, and EMT. (A) Relative mRNA expression of IL‐6, IL‐1β, IL‐10, and TNF‐α; (B) protein levels of JAK2, STAT3, and p‐STAT3 were detected by western blot; (C) protein relative expression levels compared with GAPDH; (D) the mRNA expression of E‐cadherin, Vimentin, and Collagen I by qPCR; (E–G) the expression of E‐cadherin, α‐SMA, and Collagen I in lung tissues was observed by IHC and statistical analysis of positive rate. Data are presented as mean ± SD. *p < 0.05; **p < 0.01; ***p < 0.001 compared to the Control; #*p* < 0.05; ##*p* < 0.01; ###*p* < 0.001 compared to the Model.

Inflammation is a crucial factor in the development of PF. Increased expression of cytokines, such as IL‐6, IL‐1β, IL‐10, and TNF‐α, is associated with a more intense fibrotic process (Savin et al. 2022). Therefore, we examined the mRNA transcript levels of IL‐6, IL‐1β, IL‐10, and TNF‐α in each group of lung tissues using qPCR (Figure 6A). The mRNA levels of IL‐6, IL‐1β, IL‐10, and TNF‐α were increased in the model group compared with the control group. We found that these levels decreased after EAE intervention, and the decreased expression levels of IL‐6, IL‐10, and TNF‐α showed significant differences. These data demonstrate the potential of EAE to inhibit the production of proinflammatory cytokines.

#### 3.6.2 EAE Suppresses the JAK2/STAT3 Signaling Pathway

As aberrant activation of the JAK2/STAT3 signaling pathway is closely associated with proinflammatory cytokine release and collagen synthesis during the progression of organ fibrosis (Ruan et al. 2021), we examined the expression of the JAK2/STAT3 signaling pathway proteins in fibrotic lung tissues. As shown in Figure 6B,C, the expression levels of JAK2 and p‐STAT3/STAT3 proteins were elevated in the BLM‐treated lung tissues of the mice. In contrast, EAE decreased the expression of JAK2 and p‐STAT3/STAT3 proteins. These results suggest that the anti‐inflammatory effect of EAE may be attributed to the regulation of the JAK2/STAT3 signaling pathway.

#### 3.6.3 EAE Inhibits BLM‐Induced EMT

The effects of EAE administration on PF mice were evaluated using qPCR and immunohistochemistry (IHC). The qPCR results showed that EAE significantly promoted the downregulation of vimentin and Collagen I and the upregulation of E‐cadherin at the mRNA level (Figure 6D). The IHC experiments (Figure 6E–G) obtained similar results. The administration of EAE decreased the expression of mesenchymal protein markers α‐SMA and Collagen I, as well as upregulated the expression of the epithelial cell protein marker E‐cadherin, with the changes in α‐SMA and Collagen I levels showing statistical significance. These results demonstrate that EAE may alleviate BLM‐induced PF in mice by inhibiting EMT.

## 4 Discussion

Pulmonary fibrosis is an incurable disease characterized by self‐sustaining fibrosis, extrastromal deposition, and a gradual decline in lung function (Liu et al. 2022). Given the diverse and complex pathogenesis of PF, natural products have been widely recognized as potential drugs for preventing PF because of their multicomponent and multi‐target properties (Hasan et al. 2022). It is well known that RRT is a fruit rich in nutrients and antioxidants, with multiple health benefits. Compared to previous studies on the anti‐pulmonary fibrosis effects of *Rosa roxburghii* fermentation broth (Zhou et al. 2025), this study used different solvents to extract RRT, aiming to identify the most active chemical components and integrate analyses of gut microbiota and metabolomics changes to explore its molecular mechanisms in alleviating pulmonary fibrosis. Because of the complex and diverse components of RRT, we selected four solvents with different polarities to extract its bioactive substances. Ethyl acetate is not suitable for ultrasonic extraction because of its low polarity; therefore, an extraction method was used. In this study, we found that the amount of total polyphenols, flavonoids, and triterpenes in the RRTEs was higher using EAE than when the other four extraction methods were used, indicating that the use of less polar ethyl acetate could result in the highest yield. Additionally, this method can remove more components that have no direct effects (e.g., cellulose). To accurately identify and quantify the main active compounds enriched by EAE, we made use of standardized substances for comparison. The bioactive components and their contents are closely related to their antioxidant properties. In our study, the EAE had the strongest DPPH and ABTS radical scavenging capacity when compared to the other four extraction methods, which corresponded to the highest content of bioactive substances. The strong antioxidant activity of EAE may be attributed to its rich content of active constituents, which can provide hydrogen atoms to free radicals that can then be converted into stabilized phenolic radicals, thus inhibiting a peroxidation chain reaction. These results suggest that EAE may exhibit superior health benefits, and this method was selected for subsequent experiments.

Increasing evidence supports the notion that the gut microbiota is strongly associated with lung disease and may serve as a new biomarker for the onset and progression of PF (Schuijt et al. 2016). Given that RRT is a food resource utilized by gut microbes throughout the digestive tract, we hypothesized that RRT may influence host metabolic functions by modulating the gut microbiome. Therefore, we further elucidated the changes in the gut microbiome of our PF mouse model after the EAE intervention. We found that BLM not only had a damaging effect on the lungs but also altered the composition of the gut microbiota. Higher levels of the relative abundance of harmful bacteria, such as *Ruminococcus*, *Oscillospira*, and *Bacterioides*, were found in the model group. Studies have shown that these bacteria are associated with inflammatory diseases and are more abundant in inflammatory bowel disease (Henke et al. 2021; Liu et al. 2020). The intervention of EAE inhibited BLM‐induced increases in the abundance of various pathogenic bacteria associated with inflammation and reduced bacterial inflammatory damage to tissues. EAE also increased the abundance of beneficial bacteria, including *Lactobacillus*, *Allobaculum*, *Prevotella*, *Adlercreutzia*, and *Akkermansia*, improving the structural composition of the intestinal flora. Among these bacteria, *Lactobacillus* is known to produce bacteriocins that antagonize various pathogens, including *Helicobacter* species (Prabhurajeshwar and Chandrakanth 2017; Zuo et al. 2022), thereby contributing to pulmonary protection through attenuation of oxidative stress and inflammatory responses in the lung (Zhang et al. 2022). Additionally, *Prevotella* and *Adlercreutzia* are recognized as short‐chain fatty acid (SCFA)‐producing bacteria, which exert systemic health benefits by mitigating inflammatory processes. Notably, *Verrucomicrobia* and its genus *Akkermansia* were identified as the dominant flora in the EAE‐treated group. *Akkermansia* is increasingly recognized as a next‐generation probiotic due to its role in enhancing host metabolic function and immune homeostasis. Accumulating evidence demonstrates that *Akkermansia* attenuates pro‐inflammatory cytokines, such as TNF‐α, IL‐1β, IL‐6, and IL‐33, protect intestinal and mesenteric lymph nodes, and may reduce the toxicity of sulfation‐related pathogenic bacteria through hydrogen sulfide consumption (Zheng et al. 2023), while concurrently increasing the abundance of other beneficial bacteria (Pei et al. 2023). Importantly, *Akkermansia* has been shown to ameliorate chronic renal interstitial fibrosis induced by inflammatory activation, as well as hepatic fibrosis (Pei et al. 2023; Raftar et al. 2021), suggesting a broader protective role against fibrotic pathologies across multiple organs. In this study, the EAE intervention promoted an increase in the relative abundance of *Akkermansia* in the guts of PF mice. This suggests that EAE may potentially play a positive protective role against PF conditions in the host by increasing the relative abundance of *Akkermansia* leading to specific changes in the gut microbiota.

The gut microbiota is a major regulator of host metabolism. Diet and host health affect the gut microbiota, and in turn, the composition of the gut microbiota modulates host health. This is done by their nutrient metabolizing, anti‐infective, and immune signaling capacity (Wu et al. 2021). Based on the discovery that EAE can enhance the gut microbiota of PF mice, we investigated changes in serum metabolism using metabolomics. We found that 113 metabolites were altered in mice after BLM treatment, severely affecting their normal metabolic profiles. To explore the imbalanced metabolic pathways more closely, we used a MetPA analysis of selected potential biomarkers L‐glutamate, N‐methylhistamine, L‐histidine, carnosine, L‐glutamine, pyruvate, myo‐inositol, D‐glucuronate, L‐tryptophan, 5‐HT, formylanthranilatic acid, (S)‐malate, mainly involved in histidine metabolism, D‐glutamine and D‐glutamate metabolism, phosphonate and phosphinate metabolism, alanine, aspartate and glutamate metabolism, ascorbate and aldarate metabolism, tryptophan metabolism, pyruvate metabolism. Potential biomarkers corresponding to these metabolic pathways are altered during the development of PF, which, in combination with ROC analyses, can indicate their key role in the evolution of the disease and can be considered markers for clinical diagnosis and therapy. However, we note that the predominant metabolic pathway involved in the changes during PF is the metabolism of amino acids.

Numerous studies have shown that amino acid metabolism is profoundly dysregulated in pulmonary fibrosis (PF), with elevated levels of histidine, proline, and glutamate commonly observed in PF patients (Bargagli et al. 2020; Qiu et al. 2022). Consistent with these observations, our metabolomic analysis revealed significant alterations in amino acid metabolism in bleomycin‐induced PF mice. Specifically, we observed increased levels of L‐histidine and decreased levels of carnosine and glutamine, suggesting a perturbation in histidine and glutamine metabolic networks. L‐histidine participates in multiple biological processes, including proton buffering, reactive oxygen species scavenging, and erythropoiesis (Holecek 2020). Metabolically, histidine serves as a precursor for uridylic acid and subsequently glutamine, which can be further converted into the antioxidant glutathione. Glutamine is also converted by glutaminase to glutamate, a critical step in collagen synthesis by lung fibroblasts (Hamanaka et al. 2019). In this study, EAE intervention was associated with a trend toward normalization of L‐histidine and glutamine levels in PF mice, partially reversing the BLM‐induced metabolic disturbances. Although these changes did not reach statistical significance, the observed shift toward control levels suggests that EAE may partially restore homeostasis in the histidine‐glutamine metabolic axis, potentially contributing to its anti‐fibrotic effects.

More importantly, our results showed that EAE regulated the metabolites 5‐HT and L‐tryptophan, which are involved in the tryptophan metabolic pathway, with a significant difference in the expression of 5‐HT. Our findings are similar to previous studies, in which the accumulation of tryptophan increased with the degree of lung fibrosis in mice with PF (Seo et al. 2019). Specifically, 5‐HT may promote the production of inflammatory cytokines (TNF‐α and IL‐6, among others) in PF and affect fibrotic outcomes through inflammatory processes (Redensek Trampuz et al. 2024). In contrast, 5‐methoxytryptophan (5‐MTP), the methylation product of 5‐HT (Anderson and Eighmie 2018; Wu et al. 2014), is an innate anti‐inflammatory molecule (Wu et al. 2020) that exerts anti‐PF effects by controlling multiple steps in profibroblast alterations, transcriptional reprogramming, and signaling pathways (Wu 2021). Thus, the elevated levels of L‐tryptophan and 5‐HT in PF mice may be associated with impaired tryptophan metabolism. Our EAE intervention reversed the levels of 5‐HT and L‐tryptophan induced by BLM and promoted tryptophan metabolism, indicating that EAE intervention could regulate the tryptophan metabolic pathway and 5‐HT.

Recent studies have revealed a close link between human gut microbiota, metabolites, and various physiological aspects of the host and disease progression. In modern medicine, the lungs and gut are known to be interconnected through lymphatic or blood circulation (Dang and Marsland 2019; Ma et al. 2021). Interactions between the lungs and gut can occur through circulating inflammatory cells, mediators, and metabolites (such as amino acids; Ma et al. 2021). Studies have shown that the gut microbiota can synthesize essential amino acids, promote amino acid metabolism, and maintain amino acid homeostasis (Lin et al. 2017). For example, bacteria in the genus *Clostridium* (which can utilize lysine or proline) are key drivers of amino acid fermentation, whereas bacteria in the genus *Streptococcus* are key drivers of glutamate or tryptophan use. Nevertheless, some other groups of bacteria can play a prominent role in amino acid metabolism, such as bacteria of the genera *Fusobacterium*, *Bacteroides*, and *Veillonella*, as well as *Megasphaera elsdenii* and *Selenomonas ruminantium* (Dai et al. 2011). Predictive function analysis of the differential gut microbiota in animals with lung injury disease has shown that imbalances in the gut microbiota may cause amino acid metabolism that mediates the entero‐lung axis (Lu et al. 2024).

In this study, we found that 5‐HT, a tryptophan metabolite, was positively correlated with the relative abundance of *Oscillospira* and *Bacterioides*, as well as other bacteria. The synthesis and metabolism of 5‐HT are regulated by the gut microbiota (Potter et al. 2024). Indeed, studies have found that high relative abundances of *Oscillospira* and *Bacterioides* are associated with high levels of 5‐HT (Mujagic et al. 2022). In the current study, our EAE intervention reduced the relative abundance of both *Oscillospira* and *Bacterioides*, as well as 5‐HT levels, in BLM‐induced mice compared to controls. Previous studies have demonstrated the potential role of the gut–lung axis in protecting lung health by modulating gut microbiota and metabolite destruction (Tang et al. 2024, 2023). Therefore, we hypothesized that an EAE intervention could exert a protective effect against PF by modulating the gut microbiota and mediating 5‐HT in the gut–lung axis.

5‐HT is a highly conserved and ubiquitous endogenous monoamine signaling molecule with significant effects on inflammation and immunity (Pergolizzi et al. 2022; Wu et al. 2019). During tissue injury, circulating platelets are recruited to the site of injury and activated to release 5‐HT. The subsequent increase in local 5‐HT concentration promotes pro‐inflammatory molecules and fibrotic effects, leading to enhanced tissue permeability and cytokine release (Lofdahl et al. 2020). High levels of 5‐HT and cytokines, such as IL‐6, IL‐1β, IL‐10, and TNF‐α, are frequently observed in patients with PF (Balzanelli et al. 2022; Lofdahl et al. 2023; Weng et al. 2019). IL‐6, a pro‐inflammatory cytokine produced by various cell types including fibroblasts, is closely associated with active fibrotic processes (Zhao et al. 2019). Overexpression of IL‐1β and IL‐10 has been shown to induce pulmonary collagen deposition and fibrosis (Huaux 2021; Zhang et al. 2023). TNF‐α is typically produced at high levels during the early stages of inflammation, where it induces fibroblast differentiation and recruitment of inflammatory cells, thereby amplifying inflammation through the production of cytokines such as TNF‐α and IL‐6 (Heukels et al. 2019; Yang et al. 2018). Therefore, anti‐inflammation remains a primary strategy for slowing the progression of PF and reducing disease risk. In the present study, EAE intervention significantly reduced the mRNA levels of IL‐6, IL‐1β, and IL‐10 in lung tissues. Although these measurements were performed at the late fibrotic stage (day 21 post‐BLM), persistent elevation of cytokines such as IL‐6 during the chronic phase has been documented to sustain a pro‐fibrotic microenvironment and continuously activate downstream signaling pathways (Zhao et al. 2019). Thus, the downregulation of IL‐6 mRNA by EAE at this late stage may represent a key mechanism by which EAE disrupts the IL‐6/JAK2/STAT3 positive feedback loop, thereby exerting sustained anti‐fibrotic effects.

The JAK2/STAT3 pathway is a critical signaling pathway activated by inflammatory factors (Huang et al. 2022). Growth factors such as TGF‐β1 and cytokines including IL‐6 induce JAK2 activation and phosphorylation, which in turn phosphorylates STAT3 and drives fibrotic responses (Milara et al. 2018; Zhang et al. 2015). Notably, IL‐6 serves as a classic upstream activator of the JAK2/STAT3 pathway; therefore, reduced IL‐6 levels can directly suppress STAT3 phosphorylation in lung tissue. Additionally, 5‐HT accumulation has been reported to activate the JAK2/STAT3 pathway (Banes et al. 2005). Accumulating evidence demonstrated that inhibition of JAK2 activation attenuates BLM‐induced inflammatory responses, downregulates the secretion of cytokines such as TGF‐β1, TNF‐α, IL‐1β, and IL‐6, inhibits EMT, and alleviates PF in mice (Chen, Zhao, et al. 2024; Ruan et al. 2021).

Our findings are consistent with these reports. EAE inhibited BLM‐induced activation of the JAK2/STAT3 pathway, as evidenced by reduced p‐STAT3 levels, while concurrently increasing E‐cadherin expression and decreasing the expression of vimentin, α‐SMA, and Collagen I, thereby attenuating the EMT process. Based on the observed concomitant effects of EAE on gut microbiota modulation and correction of tryptophan metabolism (5‐HT), we propose a working hypothesis: EAE may modulate gut microbiota composition, leading to improved tryptophan metabolism and reduced peripheral 5‐HT levels. Reduced 5‐HT, in turn, may suppress the production of pro‐inflammatory cytokines such as IL‐6. The consequent decline in IL‐6 levels would then inhibit JAK2/STAT3 pathway activation in lung tissue, ultimately ameliorating EMT and collagen deposition. This hypothetical framework links the macroscopic changes in the “gut‐lung axis” to local molecular events in the lung, providing a rationale for future mechanistic validation.

However, this study has certain limitations. The current data mainly reveal the association between EAE and the observed multi‐layered changes, rather than establishing definitive causal relationships. Further investigations are warranted to validate the necessity of gut microbiota remodeling through fecal microbiota transplantation experiments, to elucidate the role of 5‐HT in JAK2/STAT3 pathway activation via targeted metabolite supplementation studies, and to perform in vitro reverse validation using JAK2 inhibitors to directly assess EAE's regulatory effects on this signaling cascade.

## 5 Conclusion

In this study, we found that EAE, characterized by its abundance of bioactive constituents and potent antioxidant capacity, exerts anti‐fibrotic effects. Mechanistically, EAE intervention was associated with favorable modulation of gut microbiota composition, notably increasing beneficial taxa such as *Akkermansia* while reducing potentially pathogenic bacteria, including Bacteroides, and correcting systemic metabolic disorders, particularly tryptophan metabolism. Furthermore, EAE suppressed pulmonary inflammation and EMT via inhibition of the JAK2/STAT3 signaling pathway. In conclusion, these findings suggest that EAE exerts anti‐fibrotic effects through multi‐faceted modulation along the gut‐lung axis, involving integrated changes in microbial ecology, amino acid metabolism, and inflammatory signaling. This positions EAE as a promising candidate for further development as a preventive or therapeutic strategy for pulmonary fibrosis.

## Author Contributions

**Ting Zhou:** methodology, investigation, writing – original draft. **Heting Zhou:** methodology, investigation. **Xinyue Zheng:** methodology. **Xiaomeng Wang:** methodology. **Tao Chen:** methodology. **Xingjie Li:** methodology. **Liqun Wang:** visualization. **Shouqian Li:** methodology. **Yongmei Xie:** methodology. **Lijun Peng:** supervision, conceptualization. **Wenya Yin:** supervision, conceptualization.

## Funding

The work was supported by the Science & Technology Department of Sichuan Province, China (No. 2022NSFSC0587) and the Health Commission of Sichuan Province Medical Science and Technology Program (No. 23LCYJ009).

## Conflicts of Interest

The authors declare no conflicts of interest.

## Supporting information


**Table S1:** Primer sequences table.
**Table S2:** Possible components in RRTEs.
**Table S3:** Differential metabolites in mice serum.
**Table S4:** Location‐based metabolite sets for PF from serum Metabolomics Pathway.

## Data Availability

The data that support the findings of this study are available on request from the corresponding author.
