# Supplementary material for: The Extract of Rosa roxburghii Tratt Alleviates Pulmonary Fibrosis in Mice via Gut Microbiota‐Amino Acid Metabolism and JAK2/STAT3 Inhibition
Source: Food Sci Nutr. 2026 May 3;14(5):e71857. doi: 10.1002/fsn3.71857 (PMC13136511; doi:10.1002/fsn3.71857)
Supplement: Supplementary file 1 — Table S1: Primer sequences table. Table S2: Possible components in RRTEs. Table S3: Differential metabolites in mice serum. Table S4: Location‐based metabolite sets for PF from serum Metabolomics Pathway. [file FSN3-14-e71857-s001.docx]

**Table S1 Primer sequences table.**

| **Gene (species)** | **Sequence** | |
| --- | --- | --- |
| GAPDH（Mouse） | Forward | 5'-GGTTGTCTCCTGCGACTTCA-3' |
|  | Reverse | 5'-TGGTCCACCCTTTCTTACTCC-3' |
| IL-6（Mouse） | Forward | 5'-CTTCTTGGGACTGATGCTGGTGAC-3' |
|  | Reverse | 5'-TCTGTTGGGAGTGGTATCCTCTGTG-3' |
| IL-1β（Mouse） | Forward | 5'-ATGGTGAAGGTCGGTGTGAA-3' |
|  | Reverse | 5'-TGTCGTTGCTTGGTTCTCCTTGTAC-3' |
| IL-10（Mouse） | Forward | 5'-CGCTCTTCTGTCTACTGAACTTCGG-3' |
|  | Reverse | 5'-GTGGTTTGTGAGTGTGAGGGTCTG-3' |
| TNF-α（Mouse） | Forward | 5'-CGCTCTTCTGTCTACTGAACTTCG-3' |
|  | Reverse | 5'-GTGGTTTGTGAGTGTGAGGGTCTG-3' |
| α-SMA（Mouse） | Forward | 5'-CCTGGAGAAGAGCTACGAAC-3' |
|  | Reverse | 5'-CCCCTGACAGGACGTTGTTA-3' |
| Vimentin（Mouse） | Forward | 5'-CGTGCGGCTGCTTCAAGACTC-3' |
|  | Reverse | 5'-CTTCTCGTTGGTGCGGGTGTTC-3' |
| Collagen I（Mouse） | Forward | 5'-GACAGGCGAACAAGGTGACAGAG-3' |
|  | Reverse | 5'-ACCAGGAGAACCAGGAGAACCAG-3' |

**Table S2 Possible components in RRTEs.**

| **No.** | **Name** | **Formula** | **Molecular Weight** | **RT （min）** | **Group Area: ME/EAE** | **Group Area: WE/EAE** | **Group Area: WPE/EAE** | **Group Area: AE/EAE** | **Group Area: EAE/EAE** |
| --- | --- | --- | --- | --- | --- | --- | --- | --- | --- |
| 1 | Catechin | C15 H14 O6 | 290.07896 | 4.559 | 0.4710 | 0.6218 | 0.0130 | 0.4955 | 1.0000 |
| 2 | Procyanidin B1 | C30 H26 O12 | 578.14245 | 4.36 | 0.4747 | 0.6472 | 0.1918 | 0.4004 | 1.0000 |
| 3 | Rosamultin | C36 H58 O10 | 650.40326 | 7.42 | 2.2218 | 0.2739 | 0.0139 | 2.4017 | 1.0000 |
| 4 | α,α-Trehaloseα | C12 H22 O11 | 342.11604 | 0.819 | 3.2719 | 2.3453 | 5.0775 | 4.4274 | 1.0000 |
| 5 | Procyanidin B2 | C30 H26 O12 | 578.14246 | 4.108 | 0.2892 | 0.5019 | 0.2690 | 0.1767 | 1.0000 |
| 6 | D-(-)-Quinic acid | C7 H12 O6 | 192.06331 | 0.846 | 2.6368 | 2.8394 | 10.1666 | 2.3168 | 1.0000 |
| 7 | Ellagic acid | C14 H6 O8 | 302.00623 | 5.829 | 0.3848 | 0.3897 | 0.2585 | 0.2948 | 1.0000 |
| 8 | Citric acid | C6 H8 O7 | 192.02688 | 0.838 | 1.1499 | 3.1250 | 6.1575 | 0.7362 | 1.0000 |
| 9 | Gallic acid | C7 H6 O5 | 170.02148 | 1.812 | 0.0337 | 0.2826 | 0.0435 | 0.0433 | 1.0000 |
| 10 | Ascorbic acid | C6 H8 O6 | 176.03195 | 1.002 | 0.6987 | 0.8631 | 8.1199 | 2.1625 | 1.0000 |
| 11 | Epigallocatechin | C15 H14 O7 | 306.07389 | 3.267 | 0.2291 | 0.2572 | 0.0163 | 0.2249 | 1.0000 |
| 12 | L-Glutamic acid | C5 H9 N O4 | 147.05309 | 0.798 | 1.0076 | 2.9677 | 6.8114 | 0.9549 | 1.0000 |
| 13 | Quercetin-3β-D-glucoside | C21 H20 O12 | 464.09561 | 5.803 | 0.5405 | 0.3279 | 0.0221 | 0.5638 | 1.0000 |
| 14 | 4-Coumaric acid | C9 H8 O3 | 164.04725 | 4.555 | 0.4787 | 0.6614 | 0.0135 | 0.5108 | 1.0000 |
| 15 | D-(+)-Tryptophan | C11 H12 N2 O2 | 204.08981 | 3.906 | 2.6757 | 3.9166 | 9.1428 | 2.0364 | 1.0000 |
| 16 | Chrysin | C15 H10 O4 | 254.05781 | 8.835 | 0.8407 | 63.1812 | 0.1595 | 0.9635 | 1.0000 |
| 17 | DL-Arginine | C6 H14 N4 O2 | 174.11159 | 0.716 | 0.7455 | 2.7127 | 6.3117 | 0.2338 | 1.0000 |
| 18 | Ursonic acid | C30 H46 O3 | 454.34472 | 10.605 | 3.2624 | 1.3750 | 0.9081 | 5.2072 | 1.0000 |
| 19 | L-ascorbic acid | C6 H8 O6 | 176.03202 | 1.149 | 2.1193 | 1.8303 | 4.8752 | 2.3869 | 1.0000 |
| 20 | Trigonelline | C7 H7 N O2 | 105.02142 | 0.821 | 1.5746 | 3.4876 | 7.4095 | 0.6935 | 1.0000 |
| 21 | Galangin | C15 H10 O5 | 270.05272 | 8.959 | 0.7759 | 84.2695 | 0.4724 | 1.2764 | 1.0000 |
| 22 | Pinoresinol 4-O-glucoside | C26 H32 O11 | 520.19451 | 6.066 | 0.5006 | 0.4697 | 0.3363 | 0.5331 | 1.0000 |
| 23 | Epicatechin | C15 H14 O6 | 290.07893 | 4.904 | 0.3881 | 0.5131 | 0.0562 | 0.4475 | 1.0000 |
| 24 | Abscisic acid | C15 H20 O4 | 264.13613 | 6.849 | 0.1057 | 0.3364 | 0.0045 | 0.1423 | 1.0000 |
| 25 | Kaempferol-3-O-glucoside-6''-p-coumaroyl | C30 H26 O13 | 594.13788 | 6.91 | 22.9680 | 0.3585 | 0.1220 | 1.3821 | 1.0000 |
| 26 | (-)-epicatechin | C15 H14 O6 | 290.07895 | 5.118 | 0.2865 | 0.3226 | 0.0197 | 0.2625 | 1.0000 |
| 27 | Morin | C15 H10 O7 | 302.0427 | 7.146 | 0.0776 | 0.4131 | 0.0268 | 0.0733 | 1.0000 |
| 28 | Tyrosine | C9 H11 N O3 | 181.07385 | 1.295 | 3.3564 | 5.5143 | 15.5814 | 2.0691 | 1.0000 |
| 29 | Phenethyl caffeate | C17 H16 O4 | 284.10469 | 8.968 | 1.5383 | 129.0055 | 0.1539 | 1.7770 | 1.0000 |
| 30 | Trifolin | C21 H20 O11 | 448.10067 | 6.032 | 0.4758 | 0.3529 | 0.0265 | 0.4447 | 1.0000 |
| 31 | Taxifolin | C15 H12 O7 | 304.05831 | 6.111 | 0.0842 | 0.3840 | 0.0212 | 0.0882 | 1.0000 |
| 32 | Kaempferol-3-O-glucoside | C21 H20 O11 | 448.10069 | 6.121 | 0.8672 | 0.2210 | 0.0180 | 0.5495 | 1.0000 |
| 33 | Naringenin | C15 H12 O5 | 272.06856 | 5.773 | 0.2521 | 0.2205 | 0.0190 | 0.2657 | 1.0000 |
| 34 | Ellagic acid | C14 H6 O8 | 302.00615 | 6.294 | 0.4196 | 0.5493 | 0.6876 | 0.3410 | 1.0000 |
| 35 | 2-Hydroxy-3,4-Dimethoxybenzoic Acid | C9 H10 O5 | 198.05276 | 5.692 | 0.1884 | 0.2197 | 0.0197 | 0.1858 | 1.0000 |
| 36 | trans-Caffeic acid | C9 H8 O4 | 180.04217 | 5.005 | 0.0541 | 0.6204 | 0.0137 | 0.0909 | 1.0000 |
| 37 | Prunin | C21 H22 O10 | 434.12145 | 6.26 | 0.4593 | 0.3135 | 0.0282 | 0.5457 | 1.0000 |
| 38 | D-pantothenic acid | C9 H17 N O5 | 219.11058 | 2.93 | 0.5692 | 0.7772 | 1.3172 | 0.7253 | 1.0000 |
| 39 | Naringenin-7-O-glucoside | C21 H22 O10 | 434.1215 | 5.681 | 0.4024 | 0.5006 | 0.0945 | 0.4951 | 1.0000 |
| 40 | 2-Hydroxy-4-methylpentanoic acid | C6 H12 O3 | 132.07863 | 6.399 | 0.1383 | 0.1845 | 0.0144 | 0.1599 | 1.0000 |
| 41 | Quercetin-3-O-rhamnoside | C21 H20 O11 | 448.10135 | 5.565 | 0.6479 | 1.2390 | 3.5179 | 1.1207 | 1.0000 |

**Table S3 Differential metabolites in mice serum.**

| **No.** | **Name** | **m/z** | **rt(s)** | **M vs. C** | **EAE vs. M** |
| --- | --- | --- | --- | --- | --- |
| 1 | (-)-quebrachitol | 141.06464 | 306.9800 | * |  |
| 2 | (r)-butyrylcarnitine | 232.15387 | 252.3745 | * |  |
| 3 | 1-heptadecanoyl-sn-glycero-3-phosphocholine | 510.35225 | 185.7410 | * | * |
| 4 | 1-methylhistamine | 126.10148 | 318.8470 | * |  |
| 5 | 1-Methylnicotinamide | 137.06976 | 307.2415 | ** |  |
| 6 | 1-stearoyl-2-linoleoyl-sn-glycerol | 603.53098 | 190.8140 | * |  |
| 7 | 1,2-diamino-2-methylpropane | 72.08008 | 293.6965 | * |  |
| 8 | 1,2-dipentadecanoyl-sn-glycero-3-phosphocholine | 706.53463 | 145.5870 | * |  |
| 9 | 2-amino-1-phenylethanol | 120.08000 | 252.3690 | * |  |
| 10 | 2-aminoadipic acid | 162.07507 | 402.0925 | * |  |
| 11 | 3-aminobenzamide | 137.06946 | 283.4120 | * |  |
| 12 | 3-hydroxybutyrylcarnitine | 248.14829 | 312.7115 | * |  |
| 13 | 3-hydroxyisovaleric acid | 101.05872 | 377.7550 | * |  |
| 14 | Acetylcarnitine | 204.12199 | 298.6670 | * | ** |
| 15 | Adipoyl-l-carnitine | 290.15844 | 389.9705 | * |  |
| 16 | Ala-Thr-Arg | 174.08628 | 354.2165 | * |  |
| 17 | Decanoyl-l-carnitine | 316.24653 | 185.6070 | * | * |
| 18 | Dl-normetanephrine | 134.05922 | 29.3325 | * |  |
| 19 | DL-O-tyrosine | 146.06098 | 249.8200 | * |  |
| 20 | DL-phenylalanine | 166.08486 | 252.3615 | * |  |
| 21 | Equol | 243.10051 | 27.2635 | * |  |
| 22 | Etoposide | 606.20297 | 294.6345 | * |  |
| 23 | Gamma-glu-glu | 276.11771 | 422.8680 | * | * |
| 24 | Glu-Arg | 304.16027 | 476.9970 | ** | * |
| 25 | Glu-Ser | 235.09140 | 423.5160 | * |  |
| 26 | Glu-Thr-Arg | 203.10126 | 251.8700 | ** |  |
| 27 | Gly-Glu | 246.10761 | 385.0835 | * |  |
| 28 | Guanylurea | 103.05310 | 252.3745 | * |  |
| 29 | Hexanoyl-l-carnitine | 260.18505 | 218.3980 | * | ** |
| 30 | His-Ile | 269.15916 | 323.1730 | * |  |
| 31 | L-Glutamine | 188.10189 | 342.5310 | * |  |
| 32 | L-Histidine | 197.10228 | 357.4755 | ** |  |
| 33 | L-Lysine | 188.13824 | 573.9910 | * |  |
| 34 | L-palmitoylcarnitine | 400.33993 | 168.7575 | * |  |
| 35 | L-propionylcarnitine | 218.13780 | 274.0160 | * |  |
| 36 | L-Tryptophan | 246.12342 | 231.3450 | * |  |
| 37 | Lauroyl-l-carnitine | 344.27797 | 177.5865 | *** | ** |
| 38 | Linoleoylcarnitine | 424.33992 | 167.3650 | ** | ** |
| 39 | Marmesin | 247.08522 | 28.4855 | * |  |
| 40 | Miltefosine | 184.07202 | 174.8415 | * |  |
| 41 | Myristoyl-l-carnitine | 372.30898 | 148.3470 | * |  |
| 42 | N-(dodecanoyl)sphing-4-enine-1-phosphate | 544.41203 | 24.8755 | * |  |
| 43 | N-Acetyl-L-Histidine | 180.07618 | 357.4445 | ** |  |
| 44 | N-acetylhistidine | 198.08653 | 310.9835 | * |  |
| 45 | N6-(1-iminoethyl)-l-lysine | 188.13867 | 501.6635 | * |  |
| 46 | N6-Methyl-L-lysine | 143.11631 | 322.1515 | * |  |
| 47 | Nitrilotriacetic acid | 257.97945 | 29.5305 | * |  |
| 48 | O-acetyl-l-serine | 130.04858 | 251.8690 | * |  |
| 49 | Octanoylcarnitine | 288.21531 | 197.7670 | ** | * |
| 50 | Oleoyl-l-carnitine | 426.35523 | 165.9945 | * | ** |
| 51 | Ornithine | 174.12215 | 574.9030 | * |  |
| 52 | Phenacetine | 179.09136 | 357.4445 | ** |  |
| 53 | Pro-hyp | 229.11748 | 417.0290 | ** | ** |
| 54 | Pro-leu | 229.15354 | 360.7920 | * | * |
| 55 | Pyroglu-His-Lys | 198.12234 | 318.7740 | ** |  |
| 56 | Salannin | 619.28778 | 31.1725 | *** |  |
| 57 | Sarcosine | 131.08046 | 304.3580 | * |  |
| 58 | Ser-Thr | 207.11190 | 226.2140 | * |  |
| 59 | Serotonin | 218.12752 | 219.9635 | ** | ** |
| 60 | Thr-Ala | 191.08377 | 249.5775 | * |  |
| 61 | Trandolaprilat | 170.12721 | 501.9070 | * |  |
| 62 | Trans-cinnamic acid | 149.05825 | 252.3615 | * |  |
| 63 | trans-Vaccenic acid | 265.25116 | 58.8520 | * |  |
| 64 | Val-Ala-Lys | 159.11156 | 261.4780 | * |  |
| 65 | Valproic acid | 127.12161 | 237.8685 | * |  |
| 66 | 1-(1z-octadecenyl)-2-(4z,7z,10z,13z,16z,19z-docosahexaenoyl)-sn-glycero-3-phosphoethanolamine | 774.54237 | 134.8325 | * |  |
| 67 | 1-arachidoyl-2-hydroxy-sn-glycero-3-phosphocholine | 586.35746 | 182.1195 | * |  |
| 68 | 1-methylguanosine | 296.09829 | 192.8485 | * |  |
| 69 | 1-palmitoyl-2-linoleoyl-sn-glycero-3-phosphocholine | 802.57218 | 134.1255 | * |  |
| 70 | 1-palmitoyl-2-myristoyl-sn-glycero-3-phosphocholine | 750.53989 | 83.3840 | * |  |
| 71 | 12(R)-HETE | 319.22525 | 103.8025 | ** |  |
| 72 | 16-hydroxyhexadecanoic acid | 271.22623 | 61.5220 | * |  |
| 73 | 1h-indole-3-propanoic acid | 188.07112 | 104.8990 | * |  |
| 74 | 2-(n-morpholino)ethanesulfonic acid | 194.04791 | 105.9610 | * |  |
| 75 | 3-amino-2-(4-chlorophenyl)-2-hydroxypropanesulfonic acid | 263.99866 | 51.4095 | ** |  |
| 76 | 3-hydroxy-3-methylglutaric acid | 161.04455 | 366.4070 | * |  |
| 77 | 3-hydroxycapric acid | 187.13316 | 108.7955 | * |  |
| 78 | 3-hydroxydodecanoic acid | 215.16415 | 101.0000 | *** |  |
| 79 | 3.beta.,7.alpha.-dihydroxy-5-cholestenoic acid | 431.31265 | 101.9570 | *** |  |
| 80 | 5s,12r-dihydroxy-6e,8e,10e,14z-eicosatetraenoic acid | 335.22009 | 99.5790 | * * |  |
| 81 | All-trans-4-ketoretinoic acid | 269.21041 | 78.4120 | *** |  |
| 82 | Beta-hydroxybutyrate | 103.03968 | 230.6510 | * |  |
| 83 | Cis-7,10,13,16-docosatetraenoic acid | 331.26145 | 47.1200 | * |  |
| 84 | Cis-aconitate | 129.01859 | 433.7530 | * |  |
| 85 | D-Galactarate | 191.01888 | 117.2385 | * | * |
| 86 | D-glucuronic acid | 193.03493 | 379.6770 | *** |  |
| 87 | Dehydro-l-(+)-ascorbic acid dimer | 129.01884 | 116.6860 | ** | * |
| 88 | Dihydrojasmonic acid | 211.13277 | 102.7370 | *** |  |
| 89 | Dodecanoic acid | 199.16924 | 52.9900 | * |  |
| 90 | Eicosenoic acid | 309.27810 | 47.7270 | ** |  |
| 91 | Formylanthranilic acid | 164.03437 | 105.0060 | * |  |
| 92 | Galactonic acid | 195.05025 | 411.8450 | *** |  |
| 93 | Glutamic acid | 146.04509 | 389.7220 | * |  |
| 94 | Indole-3-carboxylic acid | 160.06077 | 401.4360 | ** |  |
| 95 | Indole-3-pyruvic acid | 202.04989 | 80.2010 | ** |  |
| 96 | Indoxyl sulfate | 212.00137 | 30.3380 | ** |  |
| 97 | Isoanhydroicaritin | 367.10273 | 381.1755 | * |  |
| 98 | L-Carnosine | 225.09821 | 411.9280 | * |  |
| 99 | Malate | 133.01393 | 405.6150 | ** |  |
| 100 | Methylmalonic acid | 117.01848 | 381.2090 | * |  |
| 101 | Myo-inositol | 179.05579 | 381.2120 | ** |  |
| 102 | Myristic acid | 227.20072 | 51.7200 | * |  |
| 103 | N-isobutyrylglycine | 144.06615 | 213.8765 | ** |  |
| 104 | N-lignoceroyl-d-erythro-sphingosine | 648.62261 | 35.4650 | * |  |
| 105 | N-palmitoyl-d-sphingosine | 536.50076 | 34.9365 | ** |  |
| 106 | Pc(18:1e/20-hdohe) | 892.59898 | 135.4630 | * |  |
| 107 | Phenaceturic acid | 192.06557 | 189.2930 | * |  |
| 108 | Pseudouridine | 243.06123 | 235.3775 | * |  |
| 109 | Pyruvate | 87.00843 | 125.7050 | * |  |
| 110 | Quillaic acid | 485.32267 | 102.9525 | * |  |
| 111 | Tetradec-5-ynoic acid | 223.16949 | 75.5540 | * |  |
| 112 | Uracil | 111.01918 | 88.4200 | * |  |
| 113 | Xanthosine | 283.06632 | 213.8445 | * * |  |

**p* < 0.05; ***p* < 0.01; ****p* < 0.001

**Table S4 Location-based metabolite sets for PF from serum Metabolomics Pathway.**

| **Pathway** | **Metabolites** | **Total** | **Hits** | **p** | **-Log(p)** | **Impact** |
| --- | --- | --- | --- | --- | --- | --- |
| Histidine metabolism | L-Glutamate; N-Methylhistamine; L-Histidine; Carnosine | 16 | 4 | 2.25E-04 | 3.6469 | 0.4016 |
| D-Glutamine and D-glutamate metabolism | L-Glutamate; L-Glutamine | 6 | 2 | 0.0058 | 2.2332 | 0.5 |
| Phosphonate and phosphinate metabolism | CMP-2-aminoethylphosphonate | 6 | 1 | 0.1175 | 0.9299 | 0.5 |
| Alanine, aspartate and glutamate metabolism | L-Glutamate; L-Glutamine;Pyruvate; | 28 | 3 | 0.0183 | 1.7383 | 0.3109 |
| Ascorbate and aldarate metabolism | Myo-Inositol; D-Glucuronate | 10 | 2 | 0.0167 | 1.7784 | 0.25 |
| Tryptophan metabolism | L-Tryptophan; Serotonin; Formylanthranilic acid | 41 | 3 | 0.0496 | 1.3041 | 0.2529 |
| Pyruvate metabolism | Pyruvate; (S)-Malate | 22 | 2 | 0.0734 | 1.1344 | 0.2379 |
